# Expanding the Use of Dynamic Electrostatic Repulsion Reversed-Phase Chromatography: An Effective Elution Mode for Peptides Control and Analysis

**DOI:** 10.3390/molecules26144348

**Published:** 2021-07-19

**Authors:** Giulia Mazzoccanti, Simone Manetto, Michele Bassan, Marco Macis, Antonia Iazzetti, Walter Cabri, Antonio Ricci, Francesco Gasparrini

**Affiliations:** 1 Department of Drug Chemistry and Technology, “Sapienza” University of Rome, 00185 Rome, Italy; simone.manetto@uniroma1.it (S.M.); francesco.gasparrini@uniroma1.it (F.G.); 2 Fresenius Kabi iPSUM, Piazza Maestri del Lavoro 7, 20063 Cernusco sul Naviglio, Italy; Michele.Bassan@fresenius-kabi.com (M.B.); marco.macis@fresenius-kabi.com (M.M.); 3 Department of Basic Biotechnological Sciences, Intensivological and Perioperative Clinics, Catholic University of Sacred Heart, 00168 Rome, Italy; antonia.iazzetti@unicatt.it; 4 Department of Chemistry, Alma Mater Studiorum-University of Bologna, Via Selmi 2, 40126 Bologna, Italy; walter.cabri@unibo.it

**Keywords:** peptide pharmaceuticals, dynamic electrostatic repulsive reversed-phase, basic analytes, high peak capacity

## Abstract

Bioactive peptides are increasingly used in clinical practice. Reversed-phase chromatography using formic or trifluoroacetic acid in the mobile phase is the most widely used technique for their analytical control. However, sometimes it does not prove sufficient to solve challenging chromatographic problems. In the search for alternative elution modes, the dynamic electrostatic repulsion reversed-phase was evaluated to separate eight probe peptides characterised by different molecular weights and isoelectric points. This technique, which involves TBAHSO_4_ in the mobile phase, provided the lowest asymmetry and peak width at half height values and the highest in peak capacity (about 200 for a gradient of 30 min) and resolution concerning the classic reversed-phase. All analyses were performed using cutting-edge columns developed for peptide separation, and the comparison of the chromatograms obtained shows how the dynamic electrostatic repulsion reversed-phase is an attractive alternative to the classic reversed-phase.

## 1. Introduction

Peptides are chemical compounds consisting of a highly variable chain of amino acids (AAs). They are considered the precursors of proteins and usually consist of a single chain of less than 50 AA units [1]. Though, peptides are interesting for their biological activity. Indeed, many endogenous hormones are peptides (such as oxytocin, insulin, and glucagon) together with some regulators of inflammation (bradykinin) and nociception (enkephalins) [2,3,4].

The first peptide used in treating disease was the hormone insulin in the 1920s [2,5,6]. Since then, peptide-based drugs have played a crucial role in clinical practice. They have also been shown to have several advantages, such as effectiveness at extremely low concentrations (due to their very high specificity) and their non-accumulation in the human body or the environment after being excreted. As a consequence of these advantages, the relevance of peptide pharmaceuticals (e.g., as antitumoral, anticoagulant, anti-hypertensive, antioxidant, antimicrobial drugs) began to increase [3,7,8,9,10].

Indeed, their use in therapy has directed several efforts on the pharmaceutical industry to research new production methods and strategies for reducing their metabolism [9,11,12,13,14,15]. As further proof of the importance of peptides in the pharmaceutical field, global industry analysis on peptide therapeutics estimated a compound annual growth rate (CAGR) of 9.1% from 2016 to 2024 retailing of peptide drugs to exceed 70 billion USD in 2019 [16]. This evolution will thrive further, given the growing incidence of metabolic diseases. Indeed, this therapeutic area represents the most relevant among all, with several glucagon-like peptides 1 (GLP-1) analogues leading the peptide market, such as Liraglutide and Semaglutide [16].

Peptide-based drugs must be analytically checked before being marketed [17]. Recently, FDA established a guide for evaluating the quality of synthetic versus recombinant peptides, which constitutes the first reference for the acceptance criteria defined for this class of products [18]. Among all the techniques reported in the guidance, evaluating the related substance content in the manufactured peptide plays a central role. The most common method to achieve this target is reversed-phase chromatography (RPC), which exploits slight differences in hydrophobicity to separate analytes of interest [19,20].

RPC is a highly flexible, robust, and reliable technique; however, it has some weaknesses. Undoubtedly the best known concerns the analysis of basic compounds, namely about 70% of pharmaceutical products [21]. The problems encountered in the analysis of basic compounds are mainly due to the complex structure of the surface of the stationary phase (e.g., free silanols, traces of metals), which result in distortions of the peak shape (i.e., tailing, asymmetry) [22,23]. Moreover, since the FDA guidance requests the evaluation of the impurity content at a very low level (e.g., 0.10% limit for unknown impurities) also for peptides, the risk they go undetected, in case they elute just after the main peak- is very high. This trend can also be detected in the biopharmaceutical field, as a growing number of pharmaceutical and biological compounds have basic properties.

Several strategies have been used to overcome these problems. Up to now, the best results have been obtained with the introduction of charged surface hybrid (CSH) packing materials [24,25,26]. These materials combine hybrid particle technology (namely, the presence of ethane bridges in the siliceous skeleton) with the presence of covalently bonded positive charges. This results in a decrease in the number of silanols (hybrid silica particles), and—working at acidic pH—it is possible to observe a repulsion between positive charges on the particle and those of protonated basic compounds. In this case, the mixture of hydrophobic interactions between the C18 chain and analyte and the repulsive ones between protonated analytes and positive charge provides a mixed-mode defined in 2014 as electrostatic repulsion reversed-phase (ERRP) [27].

Furthermore, we have tried to mimic these intermolecular interactions with commercial C18 columns using a mobile phase additive [28,29,30]. As a result, excellent outcomes have been obtained both in the analysis of low molecular weight molecules [28] and peptides [29] using tetra butyl ammonium (TBA)—such as salt—in the mobile phase. This strategy has been called dynamic ERRP (d-ERRP) in analogy to the work of 2014, as the attractive-repulsive interactions between analytes and the stationary phase are the same, with the addition of the term dynamic since they derive from the flow of the mobile phase. Similar strategies had already been developed, such as the use of ionic liquids (IL) in the mobile phase, although to our knowledge, the importance of electrostatic repulsion has never been emphasised [31,32,33]. Furthermore, the use of TBA in the mobile phase is more convenient than ILs as it is transparent to UV and requires less conditioning times. 

In this work, we want to broaden the application of d-ERRP in peptides control and analysis. For this purpose, a mixture of eight commercially available peptides used to treat various pathologies was utilised. These peptides, characterised by different molecular weight (MW) and isoelectric point (pI), were employed as probes, analysing them on different RP columns, with different characteristics, comparing mobile phases that involve the use of the most commonly used additives in RP chromatography (e.g., formic acid FA and trifluoroacetic acid TFA) with mobile phase used in d-ERRP (i.e., with TBAHSO_4_ as additive). The results were finally compared, showing the notable kinetic performance and selectivity of the d-ERRP elution mode in peptide analysis.

## 2. Results and Discussion

The d-ERRP elution mode effectively separated peptide epimers (i.e., glucagon), which was not possible with the classic additives in the RP mobile phase [29]. Therefore, given the potential of the technique, we tried to evaluate its use in the separation of eight peptides characterised by different pI (from 3.9 to 9.5) and MW (from 1096 to 4187 Da), reported in Table 1.

Two main factors can influence the separation of peptides in RPC: (i) The characteristics of the stationary phase and (ii) the composition of the mobile phase. The stationary phases used in RPC have evolved to deal with the separation of challenging compounds (often basic compounds), trying to decrease adsorption, minimising secondary interactions (e.g., with free silanols), and increasing diffusion coefficients.

Some commercially available RP columns packed with stationary phases developed to solve some of these problems were used in this work. The former group comprises columns packed with fully porous hybrid silica particles (Ethylene Bridged Hybrid, BEH) developed by Waters^®^ (i.e., (i) ACQUITY UPLC^®^ BEH C18, and (ii) ACQUITY UPLC^®^ BEH C4) [40]. The BEH particles allow reducing the number of silanols on the silica surface and having greater pH stability. Both columns are packed with particles of 300 Å pore size, suitable for peptide and protein analysis. The second type of columns are columns packed with superficially porous particles (SPP), developed by Kirkland in 2006 and marketed by Advanced technologies materials under the name of Halo [41,42,43]. Halo-ES C18 columns (in this work: (iii) Halo peptide ES-C18 (150 × 3.0 mm L × I.D.) 2.0 μm 160 Å; (iv) Halo peptide ES-C18 (150 × 4.6 mm L × I.D.) 2.7 μm 160 Å) with a pore diameter of 160 Å, effectively separate peptides, improving the kinetics of separation [44]. The anchoring chemistry of the C18 chain should also be emphasised in HALO ES-C18 columns. In fact, by exploiting the steric hindrance of the silane substituents, silanols that have remained non-derivatised are shielded without resorting to end-capping.

Regarding the elution mode, we compared RP with the most used additives, i.e., formic acid (FA) and trifluoroacetic acid (TFA), with the d-ERRP mode. The FA and TFA are used at low concentrations in the mobile phase to allow ionic pairs with the charged groups of the peptides [45,46,47].

Then, the use of mobile phases based on FA and TFA (0.1% *v*/*v*) was compared with the mobile phase d-ERRP, which involves using TBAHSO_4_ (10 mM) on all the columns described above.

Notably, the comparisons of the different mobile phases were carried out using columns of the same length, working at constant linear velocity, with the same gradient time (*t_G_*) and with columns packed with FPP 1.7 µm and SPP 2.0 and 2.7 µm particles (namely, practically identical dimensions from a kinetic point of view [44]).

This is because the work aims to evaluate the improvement, especially from the kinetic point of view, of using TBAHSO_4_ rather than the most common additives used in RPC. Thus, the evaluation was carried out by comparing the asymmetry (*As*), peak width at half height (*W*_0.5_), peak capacity *(n_c_*), and resolution (*Rs*) values, being the runs performed in gradient.

### 2.1. Comparison between IP-RPLC and d-ERRP

**Figure 1:**
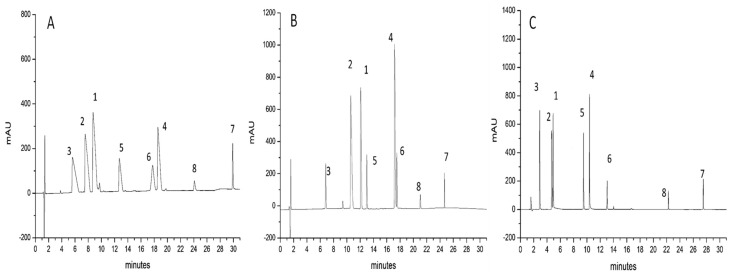
Chromatograms refer to the separation of eight peptides (see Table 1). Column used: ACQUITY UPLC^®^ BEH C18 (150 × 2.1 mm L × I.D.) 1.7 μm 300 Å FPP and BEH particle. (**A**) MP with 0.1% *v/v* FA; (**B**) MP with 0.1% *v/v* TFA; (**C**) MP with 10 mM TBAHSO_4_.

**Figure 2:**
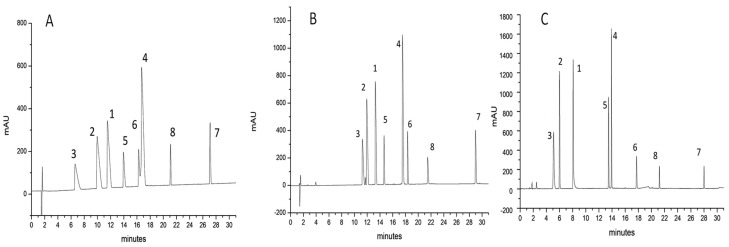
Chromatograms refer to the separation of eight peptides (see Table 1). Column used: ACQUITY UPLC^®^ BEH C4 (150 × 2.1 mm L × I.D.) 1.7 μm 300 Å FPP and BEH particle. (**A**) MP with 0.1% *v/v* FA; (**B**) MP with 0.1% *v/v* TFA; (**C**) MP with 10 mM TBAHSO_4_.

**Figure 3:**
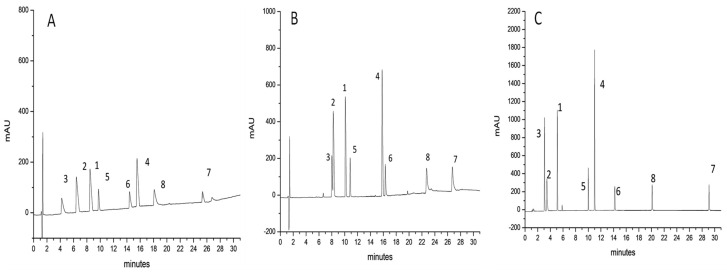
Chromatograms refer to the separation of eight peptides (see Table 1). Column used: Halo peptide ES-C18 (150 × 3.0 mm L × I.D.) 2.0 μm 160 Å SPP. (**A**) MP with 0.1% *v/v* FA; (**B**) MP with 0.1% *v/v* TFA; (**C**) MP with 10 mM TBAHSO_4_.

Figure 1, Figure 2 and Figure 3 show the separations of the eight probe peptides using FA (Figure 1A, Figure 2A and Figure 3A), TFA (Figure 1B, Figure 2B and Figure 3B), and TBAHSO_4_ (Figure 1C, Figure 2C and Figure 3C) in mobile phases, on three columns packed with different stationary phases. For example, in Figure 1, a C18 stationary phase is based on hybrid particles (BEH) FPP, in Figure 2 with C4 BEH FPP, and finally in Figure 3 with C18 SPP.

The significant difference between FA (Figure 1A, Figure 2A and Figure 3A) and TFA (Figure 1B, Figure 2B and Figure 3B) is that the formic acid leads to broader peaks and a poor peak shape since the ion pair generated is less hydrophobic [27,47,48]. The difference in terms of hydrophobicity of ionic pair become evident comparing the gradient ramps used. These have been optimised to increase the separation of the peaks on all columns using the mobile phases described. However, it is possible to observe a common trend: only with the mobile phase to which the TFA was added, the ramps start with a higher concentration of modifier organic (i.e., acetonitrile). This is not new, but it is essential to observe how, in the case of mobile phases based on TBAHSO_4_, the ramps of the developed gradients are identical to those with FA (for gradients comparison, see paragraph 3.5). However, the chromatograms shown in Figure 1C, Figure 2C and Figure 3C indicate a clear improvement in peak shape and asymmetry than those obtained with FA. This does not seem to be due to forming a strong ion pair (as in the case of TFA) but to another mechanism that allows all unwanted interactions to be reduced.

In addition to the previous observations, it is possible to note three “elution zones”, in the first one, at the beginning of the gradient, low molecular weight peptides elute (entry **3**, **2**, and **1**), in the central one, those with medium molecular weight (entry **5**, **4**, and **6**), and finally those with higher molecular weight (entry **8** and **7**). It is essential to note a change in elution order in the central area between entries **6** and **4**, which is observed using TFA and TBAHSO_4_ to FA.

The advantage in switching from the additives commonly used in IP-RP to TBAHSO_4_ in d-ERRP is observed on all columns, as can be seen in Figure S1, which shows the three chromatograms obtained in d-ERRP on the columns with stationary phase C18, whose particles of different sizes (FPP 1.7 µm and SPP 2.0 and 2.7 µm) are kinetically equivalent.

### 2.2. Asymmetry, Peak Width at Half-Height, Peak Capacity, and Resolution Values

All the analyses were performed in gradient elution. Since the classic chromatographic factors (retention factor *k*, selectivity *α*) cannot be correctly used in gradient separation [49], the separations were evaluated by examining the asymmetry (*As*), peak width at half-height (*W*_0.5_), peak capacity (*n*_c_), and resolution (*Rs*).

**Figure 4:**
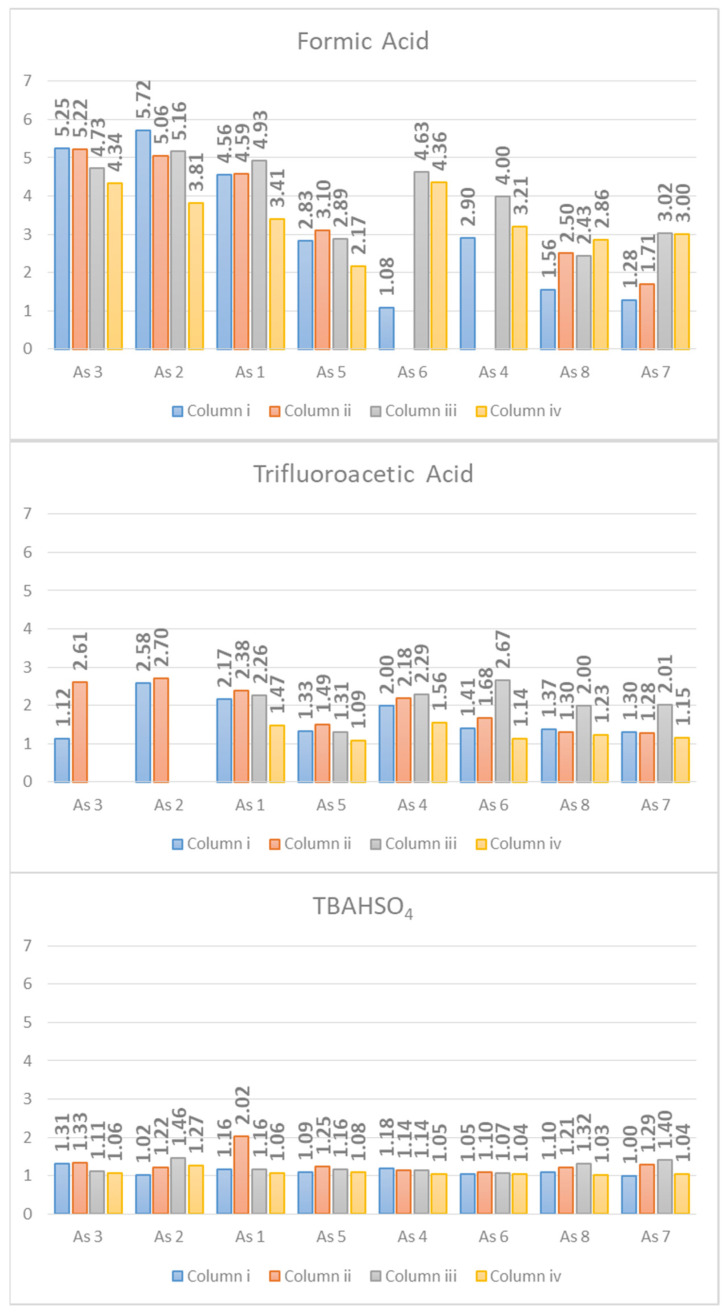
Asymmetry values (*As*).

The *As* values of each peak are reported in Figure 4, and the data were registered on all the columns used. The figure shows how the asymmetry factor drops without distinction for all the analytes on all the columns used passing from FA to TFA and further passing from TFA to TBAHSO_4_.

The peak asymmetry may be due to column overload, stationary phase heterogeneity, column packing heterogeneity or extra-column factors [50,51,52]. However, being the analysis carried out at the same experimental conditions (e.g., injection volume, chromatographic apparatus, and column temperature), and the only variable is the additive present in the mobile phase, the different adsorption of the analytes justifies this difference in the presence of a particular additive.

**Figure 5:**
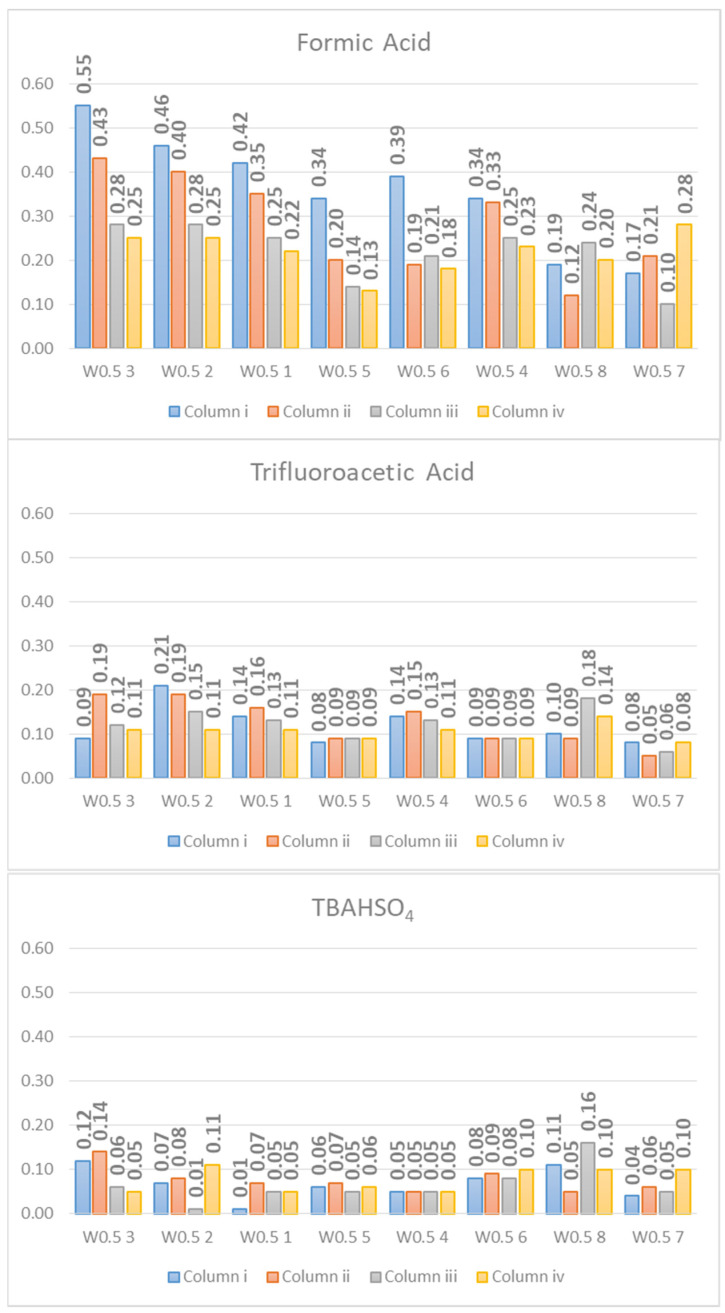
Peak Width at Half-Height (*W*_0.5_).

The peak width at half height provides information on the efficiency of the chromatographic method. Moreover, in this case, the trend shown by the bar graph (Figure 5) is clear. The *W*_0.5_ decreases passing from FA to TFA and is further reduced with TBAHSO_4_.

Another factor that considers the peak width (at the base) and that best allows us to describe the performance of a gradient separation is the peak capacity (*n_c_*) [53,54]. Peak capacity describes the maximum number of peaks that can be resolved in a gradient separation. If the peak width does not vary as a function of the retention time, it can be calculated using Equation (1).

(1) $$n_{c}=\frac{t_{G}}{W_{avg}}+1$$

**Figure 6:**
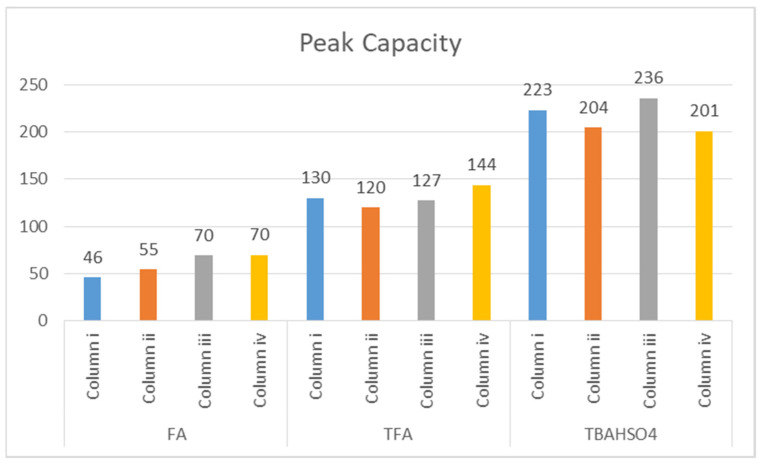
Peak capacity (*nc*) values.

With $t_{G}$ as the gradient time in minutes, and *W_avg_* as the average peak width. The gradients used lasted 30 min. In the graphs shown in Figure 6, it can be seen how the peak capacity values are higher for the separations obtained with TBAHSO_4_, with the superior value 236 for the column (iii) Halo peptide ES-C18 (150 × 3.0 mm L × I.D.) 2.0 μm 160 Å (superficially porous particles (SPP)).

In conclusion, the chromatographic factor of the resolution was evaluated. The term resolution (*Rs*) describes, in the chromatographic process, how well an analyte is separated from another, according to Equation (2):

(2) $$Rs=\frac{\sqrt{N}}{4}\;\frac{\left(\alpha -1\right)}{\alpha }\;\frac{k}{\left(k+1\right)}$$

**Figure 7:**
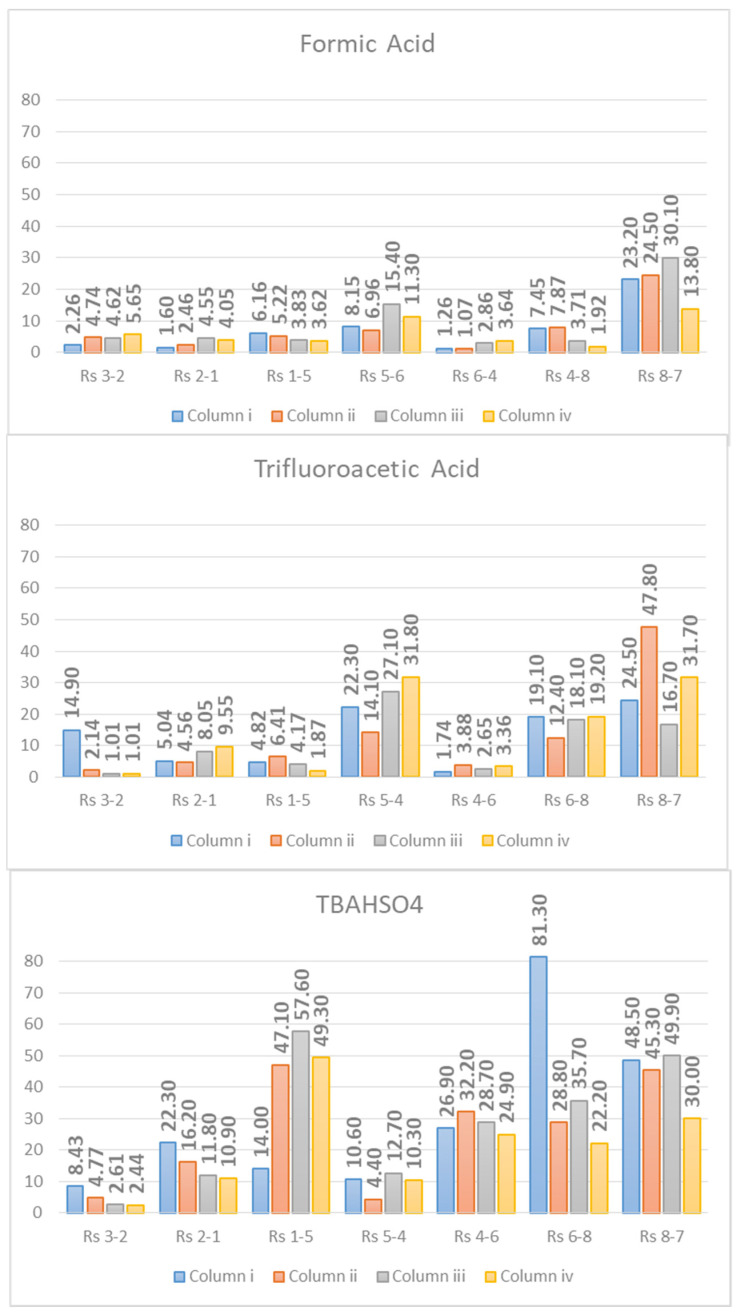
Resolution (*Rs*) values.

From the equation reported, it is clear how the *Rs* takes into account both the thermodynamic terms *k* (retention factor) and α (selectivity), and the kinetic one *N* (efficiency). It is, therefore, the complete factor for the evaluation of chromatographic separation. The *Rs* of all peak pairs using TBAHSO_4_ in the mobile phase jumped higher than that recorded for using FA and TFA (Figure 7). The *Rs* indirectly provides information on the selectivity of the method. Using TBAHSO_4_, the peaks are “better distributed” along the gradient ramp than using TFA, as demonstrated by Figure 1, Figure 2 and Figure 3.

## 3. Materials and Methods

### 3.1. Chemicals

Peptides (namely lanreotide, octreotide, icatibant, degarelix, bivalirudin, glucagon, liraglutide, semaglutide, salmon calcitonin, and exenatide) were gently given by Fresenius Kabi IPSUM (Italy). HPLC quality H_2_O and acetonitrile (ACN), tetrabutylammonium hydrogen sulfate (TBAHSO_4_) (>99% *w*/*w*), trifluoroacetic acid (TFA) (>99% *w*/*w*), and formic acid (FA) (>99% *w*/*w*) were purchased from Sigma-Aldrich (St. Louis, MO, USA). All the solvents were filtered before use on a 0.2 µm filter.

### 3.2. Sample Preparation

Each peptide stock solution was prepared at 1 mg/mL in mobile phase A (for each elution mode). Then, different volumes of each stock solution were mixed into a volumetric flask, obtaining a peptide mixture.

### 3.3. Instrumentation

UHPLC analysis was achieved using an UltiMate 3000RSLC nano-LC (Dionex, Benelux, Amsterdam, Netherlands) furnished with a binary rapid separation capillary flow pump and a ternary separation loading pump (NCP-3200RS UltiMate3000). Only the loading pump was employed in this study. The complete configuration of the system includes a thermostated column compartment and a four-channel variable wavelength detector (VWD-3400RS UltiMate 3000) with a 2.5 µL flow cell and a manual VICI Valco injector (Valco Instruments, Houston, TX, USA). The UV detector was set at a time constant of 0.10 s and a data collection rate of 100 Hz. UV detection was performed at 214 nm. The temperature of the column oven was set at 50 °C. The flow rate was set at 0.2 mL/min for those columns with 2.1 mm I.D., 0.5 mL/min for that with 3.0 mm I.D., and 1.0 mL/min for that with 4.6 mm I.D.

### 3.4. Columns

Several RP columns with different features were used:

i. ACQUITY UPLC^®^ BEH C18 (150 × 2.1 mm *L* × I.D.) 1.7 μm 300 Å (fully porous particles (FPP) and BEH (hybrid) technology particles).
ii. ACQUITY UPLC^®^ BEH C4 (150 × 2.1 mm *L* × I.D.) 1.7 μm 300 Å (fully porous particles (FPP) and BEH (hybrid) technology particles).
iii. Halo peptide ES-C18 (150 × 3.0 mm *L* × I.D.) 2.0 μm 160 Å (superficially porous particles (SPP).
iv. Halo peptide ES-C18 (150 × 4.6 mm L × I.D.) 2.7 μm 160 Å (superficially porous particles (SPP)).

### 3.5. Chromatographic Conditions

The mobile phases used in this work are the following: (1) Eluent A = H_2_O + FA (0.1% *v*/*v*); eluent B = ACN + FA (0.1% *v*/*v*); (2) eluent A = H_2_O + TFA (0.1% *v*/*v*); eluent B = ACN + TFA (0.1% *v*/*v*); (3) eluent A = H_2_O + TBAHSO_4_ 10 mM (^w^pH = 2.0); eluent B = ACN + TBAHSO_4_ 10 mM (^App^pH = 2.7); the gradients used were the following:

1. Mobile phases with formic acid as additive:
  - Column (i) gradient elution: 15% B (0 min), 15% B (1 min), 40% B (26 min), 60% B (31 min), 100% B (32 min),100% B (37 min), and 15% B (38 min).
  - Column (ii) gradient elution: 10% B (0 min), 10% B (1 min), 50% B (31 min), 100% B (32 min), 100% B (37 min), and 10% B (38 min).
  - Column (iii) gradient elution: 15% B (0 min), 15% B (1 min), 40% B (21 min), 45% B (22 min), 60% B (31 min), 100% B (32 min), 100% B (37 min), and 15% B (38 min).
  - Column (iv) gradient elution: 20% B (0 min), 20% B (1 min), 40% B (21 min), 80% B (31 min), 100% B (32 min), 100% B (37 min), and 20% B (38 min).
2. Mobile phases with trifluoroacetic acid as additive:
  - Column (i) gradient elution: 16% B (0 min), 16% B (1 min), 41% B (26 min), 61% B (31 min), 100% B (32 min), 100% B (37 min), and 16% B (38 min).
  - Column (ii) gradient elution: 12% B (0 min), 12% B (1 min), 52% B (31 min), 100% B (32 min), 100% B (37 min), and 12% B (38 min).
  - Column (iii) gradient elution: 16% B (0 min), 16% B (1 min), 41% B (21 min), 46% B (22 min), 61% B (31 min), 100% B (32 min), 100% B (37 min), and 16% B (38 min).
  - Column (iv) gradient elution: 20% B (0 min), 20% B (1 min), 40% B (21 min), 80% B (31 min), 100% B (32 min), 100% B (37 min), and 20% B (38 min).
3. Mobile phases with TBAHSO_4_ as additive:
  - Column (i) gradient elution: 15% B (0 min), 15% B (1 min), 40% B (26 min), 60% B (31 min), 100% B (32 min), 100% B (37 min), and 15% B (38 min).
  - Column (ii) gradient elution: 10% B (0 min), 10% B (1 min), 50% B (31 min), 100% B (32 min), 100% B (37 min), and 10% B (38 min).
  - Column (iii) gradient elution: 15% B (0 min), 15% B (1 min), 40% B (21 min), 45% B (22 min), 60% B (31 min), 100% B (32 min), 100% B (37 min), and 15% B (38 min).
  - Column (iv) gradient elution: 20% B (0 min), 20% B (1 min), 40% B (21 min), 80% B (31 min), 100% B (32 min), 100% B (37 min), and 20% B (38 min).

## 4. Conclusions

The d-ERRP is an effective elution mode for the separation of peptides of any molecular weight and pI. The evaluation was carried out by comparing the d-ERRP with the ion-pair reversed-phase (IP-RP), which is the most used technique for the chromatographic control of peptides and proteins. Then, the separations were carried out using mobile phases based on H_2_O and ACN with 0.1% *v/v* FA, 0.1% *v/v* TFA (IP-RP), and 10 mM TBAHSO_4_ (d-ERRP) as additives. Four columns of the same length were used, differing in particle size although kinetically equivalent (1.7 µm FPP, 2.0 and 2.7 µm SPP), and for stationary phase chemistry (column i: C18 on BEH particles, column ii: C4 on BEH particles, columns iii and iv: C18 without end-capping). All the analyses were performed using a 30-min gradient and keeping the linear velocity constant. Under these conditions, asymmetry, peak width at half-height, peak capacity, and resolution values were compared. From the analysis of these values, the superiority of the d-ERRP elution mode was demonstrated, which allowed a decrease in *As* and *W*_0.5_ and an increase in *n_c_* and *Rs* compared to IP-RP in the experimental conditions. Therefore, the d-ERRP can be used for routine control of pharmaceutical peptides, although the main drawback remains that it is not a friendly technique for mass spectrometry. Nevertheless, it can be decisive in case an improvement or a change in selectivity is required.

## Figures and Tables

**Table 1:** List of therapeutic peptides analysed. The peptides were sorted according to the length of the amino acid chain.

Entry	Peptide	Peptide Length (No. AA)	Molecular Weight (Da)	pI	Indication/Activity	Date of Market Authorisation
**1**	Lanreotide	8	1096	7.5	Acromegaly/SST agonist [34]	2007
**2**	Octreotide	8	1019	8.3	Acromegaly/SST agonist [34]	1988
**3**	Icatibant	10	1305	12.2	HHHereditary angioedema/Bradykinin B2 Receptor antagonist [35]	2008
**4**	Degarelix	10	1632	9.5	Prostate cancer/GnRH antagonist [36]	2008
**5**	Bivalirudin	20	2180	3.9	Acute coronary syndromes, Thrombotic events [37]	2004
**6**	Glucagon	29	3483	8.0	Severe hypoglycemia [38]	1962
**7**	Semaglutide	31	4113	5.4	Type 2 diabetes/GLP-1 receptor agonist [39]	2017
**8**	Exenatide	39	4187	4.9	Type 2 diabetes/GLP-1 receptor agonist [39]	2005

## Data Availability

Not applicable.

## Supplementary Materials

The following are available online. Figure S1: Chromatograms refer to the separation of eight peptides (see Table 1). MP with 10 mM TBAHSO_4_. Columns used: (**A**) ACQUITY UPLC^®^ BEH C18 (150 × 2.1 mm L × I.D.) 1.7 μm 300 Å FPP and BEH particles; (**B**) Halo peptide ES-C18 (150 × 3.0 mm L × I.D.) 2.0 μm 160 Å SPP; (**C**) Halo peptide ES-C18 (150 × 4.6 mm L × I.D.) 2.7 μm 160 Å SPP; Table S1: Asymmetry values (*As*); Table S2: Half peak width(*W*_0.5_) values; Table S3: Average peak width (W_avg_), standard deviation (STD), and peak capacity (n_c_) values; Table S4: Resolutions (*Rs*) values.

## Sample Availability

Samples of the compounds are not available from the authors.
