# Supplementary material for: Expanding the Use of Dynamic Electrostatic Repulsion Reversed-Phase Chromatography: An Effective Elution Mode for Peptides Control and Analysis
Source: Molecules. 2021 Jul 19;26(14):4348. doi: 10.3390/molecules26144348 (PMC8303375; doi:10.3390/molecules26144348)
Supplement: Supplementary file 1 [file molecules-26-04348-s001.zip › molecules-1289781-supplementary.pdf]

# Supporting information

**Giulia Mazzoccanti [1,*], Simone Manetto [1], Michele Bassan [2], Marco Macis [2], Antonia Iazzetti [3], Walter Cabri [4], Antonio Ricci [2,*] and Francesco Gasparrini [1]**

[1]Department of Drug Chemistry and Technology, "Sapienza" University of Rome, 00185 Rome, Italy; simone.manetto@uniroma1.it (S.M.); francesco.gasparrini@uniroma1.it (F.G.)
[2]Fresenius Kabi iPSUM, Piazza Maestri del Lavoro 7, 20063 Cernusco sul Naviglio, Italy; Michele.Bassan@fresenius-kabi.com (M.B.); marco.macis@fresenius-kabi.com (M.M.)
[3] Department of Basic Biotechnological Sciences, Intensivological and Perioperative Clinics, Catholic University of Sacred Heart, Rome, Italy; antonia.iazzetti@unicatt.it (A.I.);
[4]Department of Chemistry, Alma Mater Studiorum-University of Bologna, Via Selmi 2, 40126 Bologna, Italy; walter.cabri@unibo.it

**Figure S1.** Chromatograms refer to the separation of eight peptides (see Table 1 in main text). MP with 10 mM $TBAHSO_4$.Columns used: A) ACQUITY UPLC® BEH C18 (150 mm × 2.1 mm L × I.D.) 1.7 µm 300 Å FPP and BEH particle B)Halo peptide ES-C18 (150 mm × 3.0 mm L × I.D.) 2.0 µm 160 Å SPP C) Halo peptide ES-C18 (150 mm × 4.6 mm L × I.D.) 2.7 µm 160 Å SPP.

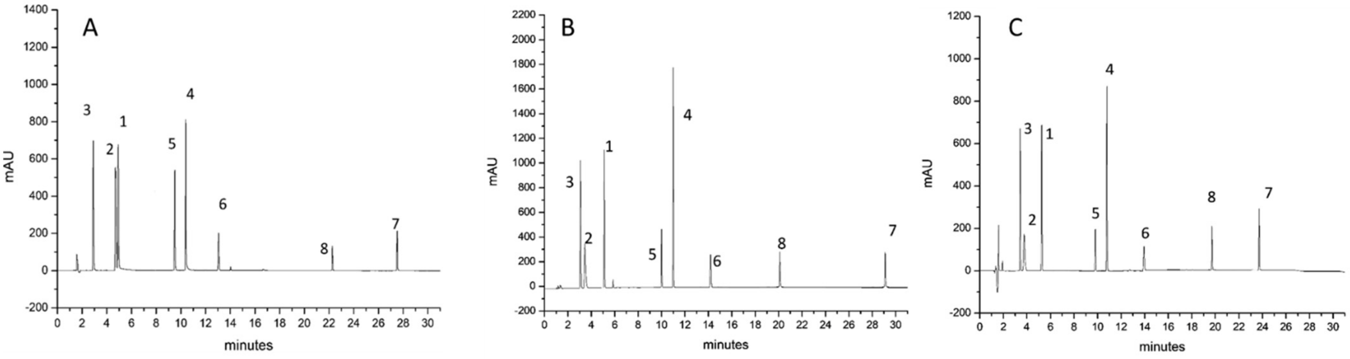


**Table S1.** Asymmetry values (As).

| | Formic Acid | | | | | Trifluoroacetic acid | | | | | TBASO$_4$ | | | |
|---|---|---|---|---|---|---|---|---|---|---|---|---|---|---|
| entry | columns | | | | en-try | columns | | | | en-try | columns | | | |
| | i | ii | iii | iv | | i | ii | iii | iv | | i | ii | iii | iv |
| **3** | 5.25 | 5.22 | 4.73 | 4.34 | **3** | 1.12 | 2.61 | n.a. | n.a. | **3** | 1.31 | 1.33 | 1.11 | 1.06 |
| **2** | 5.72 | 5.06 | 5.16 | 3.81 | **2** | 2.58 | 2.70 | n.a. | n.a. | **2** | 1.02 | 1.22 | 1.46 | 1.27 |
| **1** | 4.56 | 4.59 | 4.93 | 3.41 | **1** | 2.17 | 2.38 | 2.26 | 1.47 | **1** | 1.16 | 2.02 | 1.16 | 1.06 |
| **5** | 2.83 | 3.10 | 2.89 | 2.17 | **5** | 1.33 | 1.49 | 1.31 | 1.09 | **5** | 1.09 | 1.25 | 1.16 | 1.08 |
| **6** | 1.08 | n.a. | 4.63 | 4.36 | **4** | 2.00 | 2.18 | 2.29 | 1.56 | **4** | 1.18 | 1.14 | 1.14 | 1.05 |
| **4** | 2.90 | n.a. | 4.00 | 3.21 | **6** | 1.41 | 1.68 | 2.67 | 1.14 | **6** | 1.05 | 1.10 | 1.07 | 1.04 |
| **8** | 1.56 | 2.50 | 2.43 | 2.86 | **8** | 1.37 | 1.30 | 2.00 | 1.23 | **8** | 1.10 | 1.21 | 1.32 | 1.03 |
| **7** | 1.28 | 1.71 | 3.02 | 3.00 | **7** | 1.30 | 1.28 | 2.01 | 1.15 | **7** | 1.00 | 1.29 | 1.40 | 1.04 |

**Table S2.** Peak width at half height ($W_{0.5}$).

| | Formic Acid | | | | | Trifluoroacetic acid | | | | | TBASO$_4$ | | | |
|---|---|---|---|---|---|---|---|---|---|---|---|---|---|---|
| entry | columns | | | | en-try | columns | | | | en-try | columns | | | |
| | i | ii | iii | iv | | i | ii | iii | iv | | i | ii | iii | iv |
| **3** | 0.55 | 0.43 | 0.28 | 0.25 | **3** | 0.09 | 0.19 | 0.12 | 0.11 | **3** | 0.12 | 0.14 | 0.06 | 0.05 |
| **2** | 0.46 | 0.40 | 0.28 | 0.25 | **2** | 0.21 | 0.19 | 0.15 | 0.11 | **2** | 0.07 | 0.08 | 0.01 | 0.11 |
| **1** | 0.42 | 0.35 | 0.25 | 0.22 | **1** | 0.14 | 0.16 | 0.13 | 0.11 | **1** | 0.01 | 0.07 | 0.05 | 0.05 |
| **5** | 0.34 | 0.20 | 0.14 | 0.13 | **5** | 0.08 | 0.09 | 0.09 | 0.09 | **5** | 0.06 | 0.07 | 0.05 | 0.06 |
| **6** | 0.39 | 0.19 | 0.21 | 0.18 | **4** | 0.14 | 0.15 | 0.13 | 0.11 | **4** | 0.05 | 0.05 | 0.05 | 0.05 |
| **4** | 0.34 | 0.33 | 0.25 | 0.23 | **6** | 0.09 | 0.09 | 0.09 | 0.09 | **6** | 0.08 | 0.09 | 0.08 | 0.10 |
| **8** | 0.19 | 0.12 | 0.24 | 0.20 | **8** | 0.10 | 0.09 | 0.18 | 0.14 | **8** | 0.11 | 0.05 | 0.16 | 0.10 |
| **7** | 0.17 | 0.21 | 0.10 | 0.28 | **7** | 0.08 | 0.05 | 0.06 | 0.08 | **7** | 0.04 | 0.06 | 0.05 | 0.10 |

**Table S3.** Average peak width ($W_{avg}$) and Peak capacity ($n_c$) values.

| | Formic Acid | | | | Trifluoroacetic acid | | | | TBAHSO$_4$ | | | |
|---|---|---|---|---|---|---|---|---|---|---|---|---|
| | **i** | **ii** | **iii** | **iv** | **i** | **ii** | **iii** | **iv** | **i** | **ii** | **iii** | **iv** |
| **Peak capac-ity** | 46 | 55 | 70 | 70 | 130 | 120 | 127 | 144 | 223 | 204 | 236 | 201 |
| **$W_{avg}$** | 0.67 | 0.56 | 0.44 | 0.44 | 0.23 | 0.25 | 0.24 | 0.21 | 0.14 | 0.15 | 0.13 | 0.15 |
| **STD** | 0.29 | 0.23 | 0.13 | 0.09 | 0.09 | 0.11 | 0.08 | 0.04 | 0.07 | 0.07 | 0.09 | 0.05 |

**Table S4.** Resolution values (Rs).

| | Formic Acid | | | | | Trifluoroacetic acid | | | | | TBASO$_4$ | | | |
|---|---|---|---|---|---|---|---|---|---|---|---|---|---|---|
| entry | columns | | | | en-try | columns | | | | en-try | columns | | | |
| | i | ii | iii | iv | | i | ii | iii | iv | | i | ii | iii | iv |
| **3** | | | | | **3** | | | | | **3** | | | | |
| **2** | 2.26 | 4.74 | 4.62 | 5.65 | **2** | 14.9 | 2.14 | 1.01 | 1.01 | **2** | 8.43 | 4.77 | 2.61 | 2.44 |
| **1** | 1.60 | 2.46 | 4.55 | 4.05 | **1** | 5.04 | 4.56 | 8.05 | 9.55 | **1** | 22.3 | 16.2 | 11.8 | 10.9 |
| **5** | 6.16 | 5.22 | 3.83 | 3.62 | **5** | 4.82 | 6.41 | 4.17 | 1.87 | **5** | 14.0 | 47.1 | 57.6 | 49.3 |
| **6** | 8.15 | 6.96 | 15.4 | 11.3 | **4** | 22.3 | 14.1 | 27.1 | 31.8 | **4** | 10.6 | 4.40 | 12.7 | 10.3 |
| **4** | 1.26 | 1.07 | 2.86 | 3.64 | **6** | 1.74 | 3.88 | 2.65 | 3.36 | **6** | 26.9 | 32.2 | 28.7 | 24.9 |
| **8** | 7.45 | 7.87 | 3.71 | 1.92 | **8** | 19.1 | 12.4 | 18.1 | 19.2 | **8** | 81.3 | 28.8 | 35.7 | 22.2 |
| **7** | 23.2 | 24.5 | 30.1 | 13.8 | **7** | 24.5 | 47.8 | 16.7 | 31.7 | **7** | 48.5 | 45.3 | 49.9 | 30.0 |
